# Capillary Malformation–Arteriovenous Malformation Syndrome Associated with RASA1 and EPHB4 Mutations: Comparative Case Series and Narrative Review

**DOI:** 10.3390/life16061001

**Published:** 2026-06-14

**Authors:** Carmina Nedelcu, Catalin Cirstoveanu, Cristina Filip, Ruxandra Ioana Stefan, Ana Mihaela Bizubac, Mariana Carmen Heriseanu, Mihai C. T. Dimitriu, Nicolae Sebastian Ionescu, Mihaela Axente

**Affiliations:** 1 Neonatal Intensive Care Unit, “M.S. Curie” Children’s Hospital, Constantin Brancoveanu Boulevard, No. 20, 4th District, 041451 Bucharest, Romania; carmina.georgescu@drd.umfcd.ro (C.N.); filipcristina06@yahoo.com (C.F.); stefan.ruxandraioana@gmail.com (R.I.S.); ana-mihaela.bizubac@umfcd.ro (A.M.B.); mariana-carmen.iliescu@drd.umfcd.ro (M.C.H.); mihaela_axente@yahoo.com (M.A.); 2 Department of Neonatal Intensive Care, “Carol Davila” University of Medicine and Pharmacy, 020021 Bucharest, Romania; 3 Doctoral School, “Carol Davila” University of Medicine and Pharmacy, 020021 Bucharest, Romania; 4 Department of Pediatrics, “Carol Davila” University of Medicine and Pharmacy, 020021 Bucharest, Romania; 5 Department of Obstetrics and Gynecology, “Sf. Pantelimon” Emergency Hospital, Carol Davila” University of Medicine and Pharmacy, 020021 Bucharest, Romania; mihai.dimitriu@umfcd.ro; 6 Department of Pediatric Surgery and Orthopedics, “Carol Davila” University of Medicine and Pharmacy, 020021 Bucharest, Romania; sebastian.ionescu@umfcd.ro; 7 Romanian Academy of Medical Sciences, 030167 Bucharest, Romania; 8 Romanian Academy of Scientists, 030167 Bucharest, Romania

**Keywords:** cerebral arteriovenous malformation, CM-AVM, RASA1, EPHB4, hemodynamics, Doppler ultrasound, MRI, EEG, neonatal intensive care

## Abstract

Capillary malformation–arteriovenous malformation syndrome is a rare spectrum of vascular anomalies characterized by capillary malformations and high-flow vascular malformations, caused by loss-of-function mutations in the RASA1 and/or EPHB4 genes. These mutations disrupt vascular differentiation and lead to complex malformations involving the brain, skin, and systemic vasculature. Since the first description in 2003, more than 200 cases have been reported, but intracranial arteriovenous shunts during the neonatal period remain extremely rare, as well as reports of the dual mutation RASA1 + EPHB4 or the immunological impact of the EPHB4 mutation. We report three cases of neonates presenting with early-onset high-flow shunts, each exhibiting a distinct genetic signature: CM-AVM1 (RASA1 mutation), CM-AVM2 (EPHB4 mutation), and dual variant (combined EPHB4 and RASA1 mutations). We analyzed and compared the clinical evolution, Doppler ultrasound trends, EEG, MRI and genetic data to highlight the distinct genotype–phenotype spectrum. Early multimodal hemodynamic evaluation of neonates with CM-AVM allows the identification and optimum management of life-threatening shunts at the earliest possible stage.

## 1. Introduction

Capillary malformations typically present as macular lesions and represent slow-flow vascular anomalies composed of dilated and/or numerically increased dermal capillaries [1]. The association between cutaneous capillary malformations, high-flow arteriovenous shunts, and heterozygous mutations in the RASA1 gene was first reported by Eerola et al. in 2003 [1] as the capillary malformation–arteriovenous malformation syndrome. Amyere et al. [2] refined the nomenclature in 2017, designating phenotypes caused by RASA1 pathogenic variants as capillary malformation–arteriovenous malformation 1 (CM-AVM1) and those arising from EPHB4 pathogenic variants as capillary malformation–arteriovenous malformation 2 (CM-AVM2). These mutations produce loss of function of the encoded proteins, resulting in hyperactivation of the RAS–RAF–MEK–ERK pathway, leading to significant clinical overlap between CM-AVM1 and CM-AVM2, although phenotypic distinctions exist.

CM-AVM syndrome follows an autosomal dominant inheritance pattern and exhibits high penetrance for cutaneous lesions. The estimated prevalence in Northern European populations is approximately 1:100,000 [3]. Analyses of the Exome Aggregation Consortium dataset report a higher prevalence of approximately 1:20,000 for CM-AVM 1 and 1:12,000 for CM-AVM 2 [2].

Clinically, CM-AVM is characterized by multiple small capillary malformations, predominantly involving the face and limbs. Some individuals also develop arteriovenous malformations (AVMs) or arteriovenous fistulas (AVFs) in the brain, spine, muscle or bone. These lesions can lead to life-threatening complications, including hemorrhage, congestive heart failure, or neurological deficits. Intracranial AVMs/AVFs frequently manifest early in life, and some affected individuals may present with Parkes Weber syndrome [4].

An arteriovenous malformation is a high-flow vascular anomaly characterized by multiple direct arterial–venous connections, producing a low-resistance shunt and dilation of adjacent feeding arteries and draining veins. Cerebral high-flow arteriovenous shunts represent rare intracranial vascular malformations that may involve choroidal, pial or dural compartments. Classification is based on their anatomical location, angioarchitecture, and age at clinical presentation. In neonates and young infants, symptoms typically arise from circulatory compromise due to significant arteriovenous shunting [5], while later presentations frequently involve seizures or intracranial hemorrhage. Early onset is generally associated with a poor prognosis; however, advances in early diagnosis and endovascular intervention have substantially improved outcomes.

This report presents three neonatal patients with distinct high-flow vascular anomalies—two of them with cerebral arteriovenous malformations—accompanied by cutaneous capillary malformations, and one case associated with secondary hemophagocytic lymphohistiocytosis.

## 2. Case Reports

### 2.1. Case 1—RASA1 Mutation (CM-AVM1)

A term, 1-month-old male infant was referred to the Neonatal Intensive Care Unit for evaluation of suspected partial anomalous pulmonary venous return and dilated right-sided cardiac cavities. Physical examination identified a systolic murmur, superficial telangiectatic lesions over the left parietal scalp, a pale pink macule (1 × 2 cm) in the occipital region, accompanied by an accentuated cranial bruit in the anterior fontanelle, which was otherwise normotensive, as well as mild hypertonia of the right limbs. The cardiovascular status was otherwise stable.

Echocardiography and vascular Doppler imaging excluded anomalous pulmonary venous return and confirmed the dilation of the right cardiac cavities, bovine aortic arch, ectasia of the brachiocephalic arterial trunk and main pulmonary artery, as well as marked tortuosity of the left internal carotid artery with predominantly turbulent flow. These findings prompted consideration of arterial tortuosity syndrome (characterized by tortuosity of the aorta and/or mid-sized arteries, focal stenosis of segments of the pulmonary arteries) or an underlying connective tissue disorder (characterized by multiple unusual vascular findings in different arterial territories).

**Figure 1:**
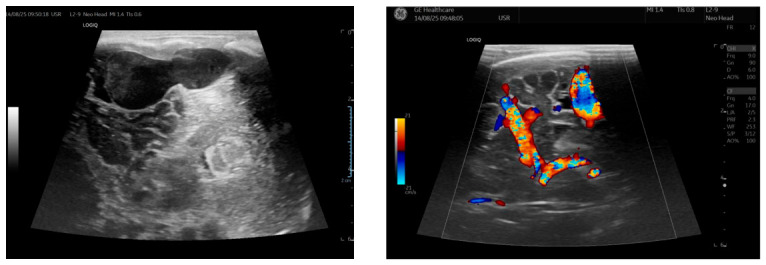
Cranial ultrasonography and transcranial Doppler show the arteriovenous malformation nidus—significantly enlarged temporo-occipital vessels with turbulent flow, suggestive of an AVM.

Cranial ultrasonography identified an arteriovenous malformation nidus in the left temporo-occipital region, characterized by turbulent flow, asymmetric dilation of the bilateral sigmoid and transverse venous sinuses—more pronounced on the left—and atrophy of the left cerebral hemisphere, accompanied by a small left frontal subdural collection (Figure 1).

**Figure 2:**
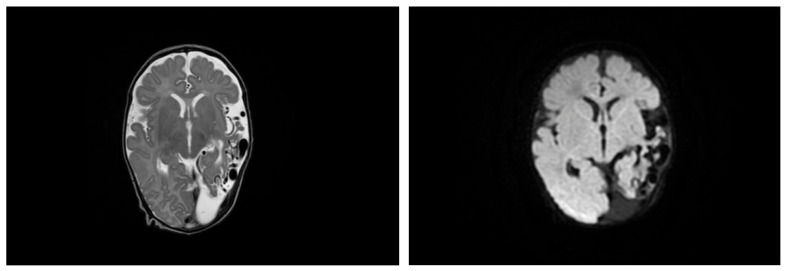
Axial T2 and 3D time-of-flight (TOF) sequences illustrate severe parenchymal atrophy and significant steal effect in the adjacent cortex, in settings of brain AVM.

Brain MRI further delineated a complex vascular malformation within the left temporo-parieto-occipital lobe (nidus diameter—30 mm), consisting of tortuous vascular channels linking the deep arterial circulation (distal internal carotid artery and basilar artery) with the pericerebral venous system. The left pericerebral veins were dilated and tortuous, draining into a proximally saccular and enlarged left sigmoid sinus. The intracranial vertebral arteries also appeared tortuous and dilated. Moderate to severe parieto-occipital atrophy was observed. Additionally, the entire course of the left common carotid artery displayed mildly increased caliber with distal tortuosity (Figure 2).

**Figure 3:**
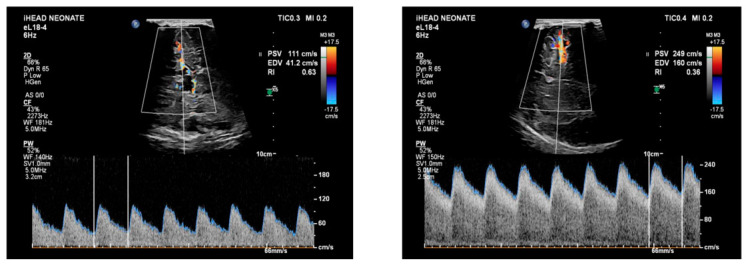
Transcranial Doppler ultrasound shows increased systolic (PSV) and diastolic velocities (EDV) and a low resistivity index (RI) in the right middle cerebral artery and perforating artery.

Hemodynamic monitoring through serial transcranial Doppler examinations demonstrated a substantial increase in systolic flow velocity and a progressive decline in vascular resistance (middle cerebral artery: PSV = 111 cm/s, RI = 0.63; perforating artery: PSV = 249 cm/s, EDV = 160 cm/s, RI = 0.36) (Figure 3). This hemodynamic profile was consistent with a high-flow, low-resistance shunt, characteristic of an intracerebral arteriovenous malformation.

The MRI flow-void cluster corresponded anatomically to the region of maximal Doppler acceleration, confirming the concordance between hemodynamic and structural data. No diffusion restriction or intracranial hemorrhage were identified, suggesting chronic but compensated cortical hypoperfusion consistent with a steal phenomenon. All these findings were diagnostic of a congenital high-flow arteriovenous malformation associated with severe parenchymal atrophy and a significant steal effect in the adjacent cortex.

**Figure 4:**
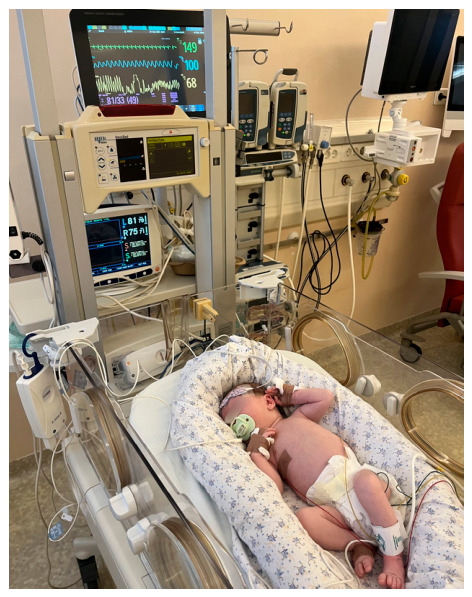
Continuous multimodal hemodynamic monitoring—heart rate, SpO_2_, blood pressure, respiratory rate, NIRS (ScO_2_)—according to our unit’s protocol for noninvasive hemodynamic monitoring [6].

Cerebral perfusion assessed via near-infrared spectroscopy (NIRS) remained within the normal range (right frontal ScO_2_ = 75%; left frontal ScO_2_ = 80%) (Figure 4).

**Figure 5:**
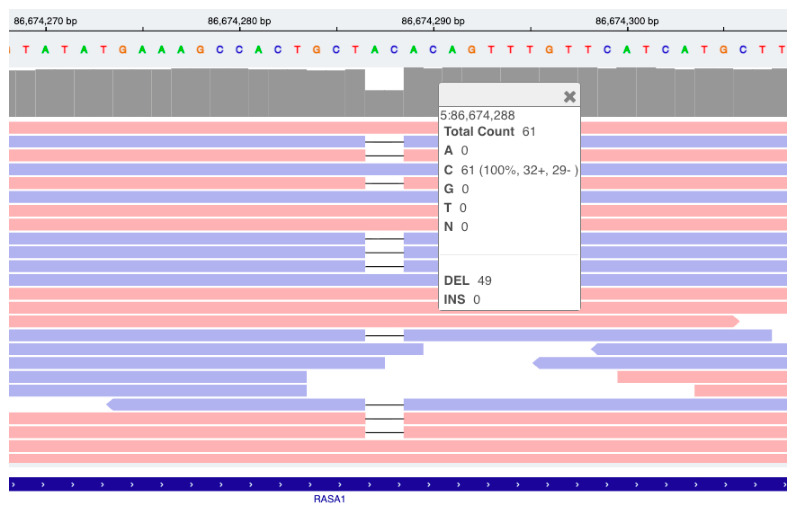
Integrative Genomics Viewer (IGV) screenshot of the pathogenic *RASA1* variant identified by whole exome sequencing. The heterozygous deletion c.2422_2423del, p.(Gln808Valfs*21) is visible as aligned reads containing a two-base pair deletion, resulting in a frameshift and premature termination codon. Reproduced with permission from Blueprint Genetics.

Whole-exome sequencing completed the picture, establishing the diagnosis of CM-AVM1 by identifying a heterozygous frameshift variant in the RASA1 gene, c.2422_2423del (p.Gln808Valfs*21), classified as likely pathogenic (Figure 5).

Given the patient’s stable clinical condition despite the presence of cranial arterio-venous malformation and the absence of systemic hemodynamic compromise, he was discharged home and scheduled for continued outpatient follow-up until definitive AVM embolization could be performed.

### 2.2. Case 2—EPHB4 Mutation (CM-AVM2)

A full-term male neonate from a partially monitored pregnancy (without prenatal anomalies) developed severe respiratory distress immediately after birth requiring resuscitation and invasive mechanical ventilation. He was referred to our Neonatal Intensive Care Unit for severe pulmonary hypertension and suspected congenital heart disease.

**Figure 6:**
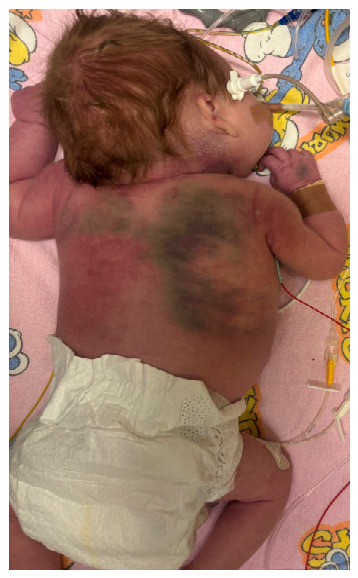
An evolving clinical picture characterized by hemihypertrophy of the left upper limb and migratory erythematous–violaceous cutaneous lesions with ecchymoses, predominantly involving the upper extremity.

Clinical findings on admission included scalp and upper thoracic edema, left-sided hemihypertrophy with approximately 2 cm limb circumference difference between the arms, and mild erythema over the left thoracic wall (Figure 6). The infant was hypoactive but demonstrated symmetrical distal limb movements in response to stimulation.

**Figure 7:**
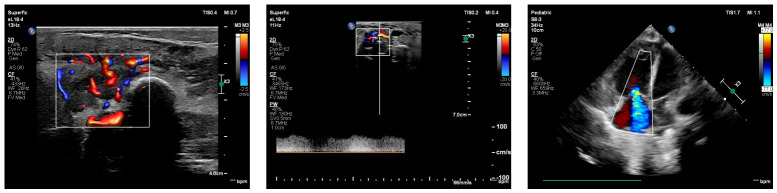
Left arm soft tissue Doppler ultrasound—well-visualized vascular network with systolo-diastolic flow. Significant right cardiac cavities dilatation and severe tricuspid regurgitation, in settings of severe pulmonary hypertension.

Echocardiography revealed severe pulmonary hypertension with enlarged right cavities, severe tricuspid regurgitation, and increased flow in the superior vena cava (Figure 7). Soft tissue Doppler ultrasound of the left arm showed a diffuse, well-visualized vascular network with systolo-diastolic flow corresponding to the most hypertrophied left arm area (Figure 7).

**Figure 8:**
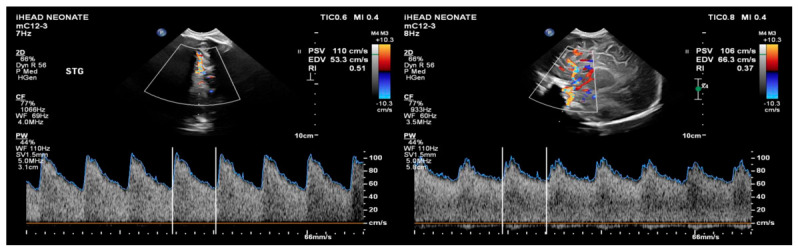
Transcranial Doppler imaging on the anterior cerebral artery (ACA)—progressive increase in diastolic velocities with a decrease in RI.

Serial transcranial Doppler imaging (anterior cerebral artery, middle cerebral artery, venous sinuses) revealed progressive bilateral hemodynamic changes—PSV increased from 48.8 to 106 cm/s, EDV increased from 30.9 to 38.9 cm/s, while RI markedly decreased from 0.80 to 0.34, predominantly driven by an increase in diastolic velocities (30.9 → 66.3 cm/s) (Figure 8). These markedly reduced resistive indices reflected profound cerebral vasodilatation and loss of autoregulatory control. The progressive decline in RI coincided with clinical worsening (systemic arterial hypotension, especially low diastolic pressure) and metabolic imbalance.

**Figure 9:**
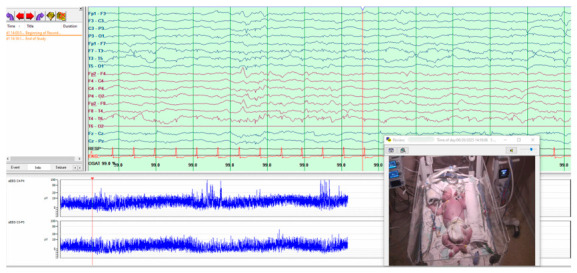
aEEG/cEEG—discontinuous, low voltage pattern.

A 2 h aEEG/cEEG monitoring showed a low-voltage, diffuse, symmetric delta continuous pattern, without reactivity or variability (Figure 9). The aEEG tracing was discontinuous (4–15 µV), lacking sleep–wake cycles, compatible with diffuse cortical depression under sedation but also suggestive of neuronal disfunction.

Combined Doppler ultrasound and EEG data indicated diffuse vascular dysregulation. Markedly increased diastolic cerebral blood flow velocities reflected profound vasodilation and loss of autoregulation. Despite preserved systolic flow, cerebral perfusion became pressure-dependent and inefficient at the microcirculatory level, leading to metabolic neuronal dysfunction. EEG abnormalities therefore reflected functional impairment due to perfusion–metabolism mismatch rather than structural injury or cerebral edema.

Thoracic CT angiography demonstrated a dilated, tortuous subclavian artery and an anomalous vessel originating from the aortic arch with distal drainage into an arteriovenous malformation which could not be fully characterized based on the acquired images.

Laboratory evaluation showed a severe inflammatory activation pattern—markedly elevated ferritin (185.303 ng/mL), interleukin-6 (286.5 pg/mL), aminotransferase, triglyceride and creatinine levels, associated with thrombocytopenia, but with C-reactive protein and procalcitonin within the normal range. The extreme hyperferritinemia and marked IL-6 elevation, in the absence of infectious markers, supported a non-infectious inflammatory process consistent with macrophage activation syndrome (MAS) or secondary hemophagocytic lymphohistiocytosis (HLH).

The patient developed severe deterioration and rapidly progressive clinical course over the last 48 h, culminating in death at less than 24 h after HLH diagnosis was established, despite maximal supportive therapy and corticosteroid therapy.

**Figure 10:**
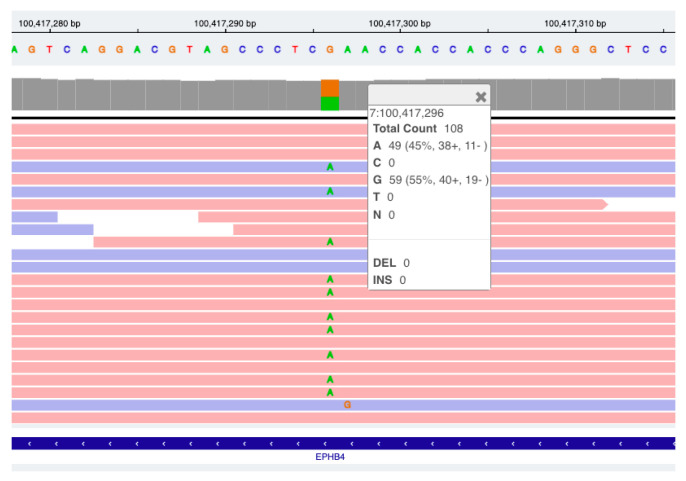
Integrative Genomics Viewer (IGV) screenshot of the pathogenic *EPHB4* variant identified by whole exome sequencing. The heterozygous nonsense variant c.1180C > T, p.(Arg394*) is supported by approximately 55% of aligned sequencing reads at the corresponding genomic position, compatible with heterozygous allelic distribution. Reproduced with permission from Blueprint Genetics.

Genetic testing identified a heterozygous pathogenic EPHB4 variant—c.1180C > T, p.(Arg394*), confirming capillary–arteriovenous malformation type 2 (CM-AVM2) (Figure 10).

**Figure 11:**
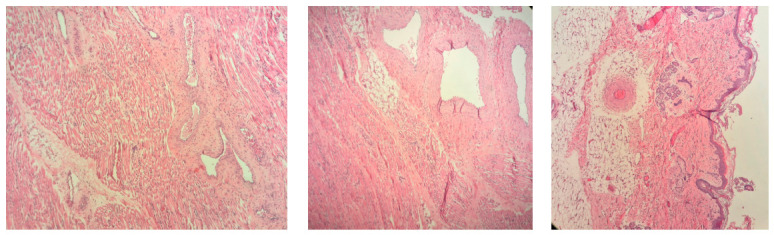
Histiocytic infiltration with hemophagocytosis in the bone marrow. Hematoxylin and eosin (H&E) staining, original magnification ×100.

The anatomopathological report was compatible with active hemophagocytic syndrome: histiocytic infiltration with hemophagocytosis in bone marrow and reticuloendothelial tissues, endothelial disruption and microvascular hemorrhage (Figure 11). These observations confirmed macrophage activation superimposed on a background of EPHB4-related vascular fragility.

### 2.3. Case 3—Dual EPHB4 + RASA1 Mutation

A term female neonate from a pregnancy complicated by hydrops fetalis with polyhydramnios and mild pericardial effusion, was delivered by emergency due to non-reassuring fetal heart rate. At birth the neonate was hypotonic, cyanotic, and required prolonged resuscitation and mechanical ventilation, with an Apgar score of 2/6 at 1/5 min, respectively. Physical examination revealed diffuse facial telangiectatic lesions involving the right fronto-orbital and zygomatic region, a violaceous reticular capillary pattern on the trunk, and macrocephaly with facial asymmetry/hypertrophy. No cardiac murmurs or limb hypertrophy were noted. Neurological evaluation [7] showed generalized hypotonia, depressed neonatal reflexes, and spontaneous clonic movements of the right arm. Continuous sedation was required due to irritability and respiratory instability.

Cranial ultrasound on the first day of life revealed increased echogenicity of periventricular white matter, asymmetric venous sinuses, and mildly dilated lateral ventricles. Cardiac ultrasound showed normal intracardiac anatomy, with no evidence of high-output cardiac failure. The EEG recording on the fifth day of life was compatible with global cortical dysfunction due to hypoperfusion and sedation—low-amplitude discontinuous trace with background of 20–25 µV.

**Figure 12:**
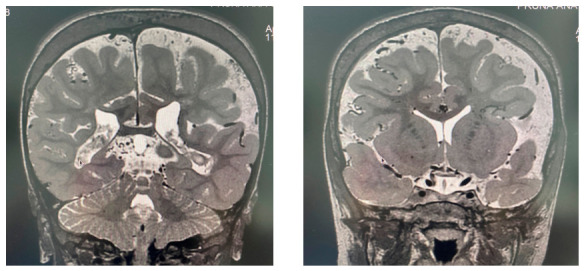
Brain MRI findings were consistent with a congenital high-flow arteriovenous malformation associated with venous dysplasia and secondary parenchymal hypoperfusion.

Brain MRI (T1/T2/FLAIR/DWI sequences) showed marked thickening of the cranial vault, most prominent in the parietal–occipital regions, suggesting chronic osseous remodeling secondary to longstanding hyperemia. Multiple serpiginous flow voids within the subdural and cortical spaces indicated the presence of abnormal vascular channels or shunts (Figure 12). In addition, there were dilated venous structures coursing along the subdural plane and superficial cortical surfaces. Subtle white matter signal changes were suggestive of chronic venous congestion or mild hypoxic injury, without acute hemorrhage.

**Figure 13:**
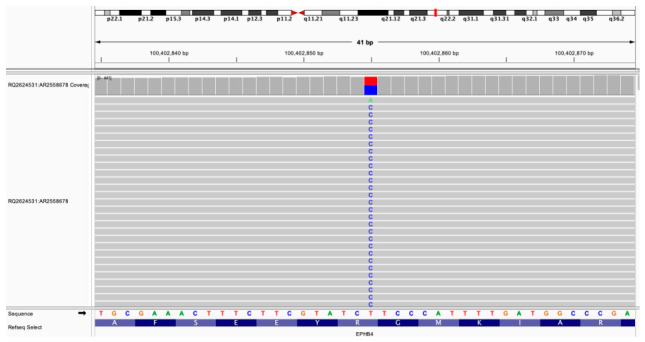
IGV visualization of the heterozygous pathogenic variant EPHB4 c.1219C > T (p.Arg407*) located on chromosome 7. The variant is supported by approximately 50% of sequencing reads, consistent with a heterozygous genotype. This nonsense variant introduces a premature termination codon, predicted to result in truncation of the EPHB4 receptor protein and loss of function. Reproduced with permission from Invitae/Labcorp.

**Figure 14:**
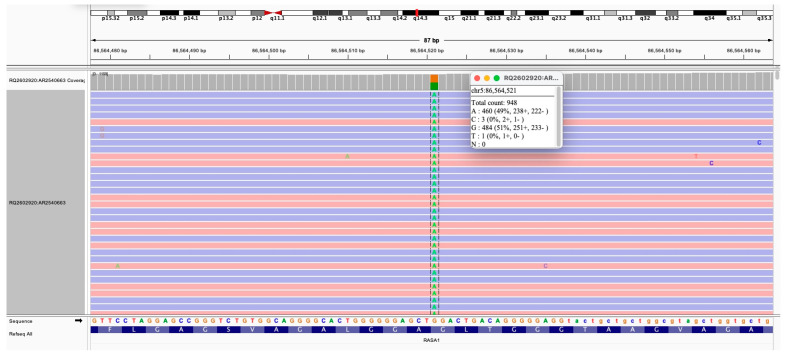
IGV visualization of the heterozygous pathogenic variant RASA1 c.1427delA (p.Lys476Serfs*5) located on chromosome 5. The deletion is present in approximately half of sequencing reads, consistent with a heterozygous genotype. This frameshift variant is predicted to generate a premature stop codon after five altered amino acids, resulting in truncation of the p120-RasGAP protein. Reproduced with permission from Invitae/Labcorp.

Comprehensive molecular testing revealed two pathogenic variants: EPHB4 c.1219C > T (p.Arg407*)—nonsense variant, predicted to result in truncated receptor protein and loss of function, and RASA1 c.1427delA (p.Lys476Serfs*5)—frameshift deletion, leading to premature truncation of p120-RasGAP (Figure 13 and Figure 14). Both variants were heterozygous and de novo, confirming a dual-gene vascular signaling defect.

Histopathology (skin biopsy) showed irregular capillary and venular channels within the endothelium and disrupted perivascular architecture, absence of elastic lamina, supporting a diagnosis of combined capillary–arteriovenous malformation.

At follow-up, the infant showed a relatively stable clinical course. During the first years of life, motor development was mildly delayed, with persistent hypotonia and slower acquisition of early motor milestones. Cognitive development was also moderately delayed, but gradual improvement in environmental awareness and interaction with caregivers was noted. No clinical seizures were reported, and there were no episodes of acute neurological deterioration. This evolution supported the interpretation of a chronic, predominantly hemodynamic developmental encephalopathy related to the congenital high-flow arteriovenous malformation, venous dysplasia, and secondary cerebral hypoperfusion, rather than an epileptic or rapidly progressive neurological disorder.

## 3. Literature Review

### 3.1. Pathophysiology

Capillary malformation–arteriovenous malformation (CM-AVM) syndrome is mostly inherited in an autosomal dominant manner, although de novo pathogenic variants have been reported. To date, two genes have been identified to be implicated: RASA1—responsible for CM-AVM type 1, and EPHB4—responsible for CM-AVM type 2. Together, germline variants in RASA1 and EPHB4 explain approximately 50% and 25% of affected individuals, respectively [8]. The remaining cases are likely attributable to alternative genetic mechanisms, such as genomic rearrangements, which have been detected in 8.6% of patients with CM-AVM1 [9].

Current evidence supports a two-hit pathogenic mechanism in the development of CM-AVM [10,11,12]. In this model, a germline heterozygous pathogenic variant constitutes the first hit, while a somatic post-zygotic mutation in endothelial cells leads to complete loss of function locally. This biallelic inactivation is required for the formation of both capillary malformations and high-flow vascular lesions [12,13]. Somatic second-hit mutations in RASA1 have been identified in tissue samples, confirming that loss of function of both alleles facilitates vascular lesion development [11]. Similar mechanisms have been described in other inherited vascular malformation syndromes with multifocal presentations, including glomuvenous malformations and cerebral cavernous malformations [14,15,16,17].

The timing and location of the second-hit mutation contribute to the clinical heterogeneity of CM-AVM [18]. Early post-zygotic events, potentially occurring in utero, are thought to result in arteriovenous malformations and explain their early clinical manifestation, whereas later somatic mutations may lead to the delayed appearance of capillary malformations [10]. This also explains the marked intrafamilial variability observed regarding lesion number, distribution, and the presence/absence of severe arteriovenous shunts.

Most RASA1 pathogenic variants associated with CM-AVM are frameshift or deletion mutations that introduce premature stop codons, resulting in truncated proteins with complete loss of function of the Ras-GTPase Activating Protein (RasGAP) domain [1]. Haploinsufficiency of RASA1, combined with somatic loss of the remaining functional allele, leads to aberrant Ras signaling and formation of high-flow vascular lesions [10]. RASA1 encodes p120RasGAP, a widely expressed negative regulator of Ras activity that plays a critical role in controlling endothelial cell proliferation, migration, polarity, and angiogenesis. Dysregulation of Ras signaling affects multiple angiogenic mechanisms by hyperactivating VEGF signaling, suppressing antiangiogenic glycoproteins, and upregulating extracellular matrix-degrading protease [19].

RASA1 functions downstream of EPHB4, a transmembrane receptor tyrosine kinase. EPHB4 and its ligand, ephrin-B2, which is expressed on arterial endothelial cells, form a bidirectional signaling system that cooperates with Notch signaling to regulate arterial–venous differentiation and vascular remodeling [20]. Activation of EPHB4 recruits intracellular RASA1, leading to suppression of the RAS-MAPK-ERK1/2 and PI3K-AKT-mTORC1 signaling pathways [2]. Inactivating variants in either EPHB4 or RASA1 disrupt this regulatory axis, resulting in constitutive activation of downstream signaling cascades, overactivation of mTORC1, and vascular overgrowth [20]. Recent studies have suggested that EPHB4 dysfunction can extend beyond structural malformations, affecting vascular tone, endothelial integrity, and inflammatory signaling [21].

The phenotypic similarity between CM-AVM1 and CM-AVM2 highlights the shared molecular pathway involving EPHB4–RASA1 signaling and downstream Ras-dependent effectors. Integration of genotypic-phenotypic data has important clinical implications—it may aid in refining classification of vascular malformations and in guiding management strategies, particularly in complex cerebrovascular lesions. For example, RASA1 variants are associated with a broader range of vascular malformations—including AVMs, pial AVF, and vein-of-Galen malformation—whereas EPHB4 variants appear to be specific to vein of Galen aneurysmal malformations, underscoring the value of molecular diagnostics in personalized patient care [22,23].

### 3.2. Clinical Characteristics

The clinical spectrum of CM-AVM has been extensively characterized (Table 1), particularly for RASA1-associated CM-AVM through large cohort studies [1,3,10], as well as numerous case series and reports. In contrast, the phenotype of EPHB4-associated CM-AVM has been described in fewer studies [2,24]. A hallmark of CM-AVM syndrome is the marked intra- and interfamilial phenotypic variability.

In CM-AVM1, capillary malformations are present in approximately 97% of individuals, whereas arteriovenous malformations or fistulas occur in nearly one quarter, involving extracranial sites (13%) or the central nervous system (10%) [10]. Nonetheless, the true prevalence of high-flow lesions is likely underestimated, as many patients have not undergone systematic imaging. Parkes Weber syndrome is reported in approximately 8% of patients, while Bier spots and telangiectasias are uncommon [10].

Capillary malformations constitute the most common and often earliest clinical sign of CM-AVM and are critical for early diagnosis. These lesions are typically multifocal, small (1–2 cm), round-oval, pink-to-red or red-to-brown macules, frequently surrounded by a characteristic pale halo suggestive of the steal phenomenon [9]. They most commonly involve the face, trunk, and extremities, may be present at birth and increase in number progressively over time [4,25]. Larger, irregular, or confluent capillary malformations, sometimes referred to as “herald patches”, are often associated with underlying high-flow vascular malformations [26]. In darker skin phototypes, brownish CMs may be misdiagnosed as café-au-lait macules.

Some of these cutaneous vascular lesions demonstrate clinical and/or imaging features suggestive of underlying arteriovenous shunting, including elevated skin temperature, palpable thrill, or Doppler pulsatility, reflecting the arterial component [27]. Ultrasonographic studies have shown increased blood flow, dilated vessels, and fast-flow signals beneath these cutaneous lesions, particularly those larger than 1.5 cm [3,12,28]. The detection of dermal or subcutaneous fast-flow anomalies beneath capillary malformations should prompt consideration of a RASA1 or EPHB4 mutation and warrants screening for extracutaneous AVMs.

High-flow vascular malformations in CM-AVM syndrome may involve the skin, subcutaneous tissues, muscle, bone, extremities, neck, spine, and central nervous system. Unlike hereditary hemorrhagic telangiectasia (HHT), visceral involvement of the lungs or liver was extremely rarely reported. Yakes et al. [29] proposed a classification based on the angioarchitecture: type I, arteriovenous fistula (AVF)—direct connection between an artery and a vein; type II, arteriolovenous malformation or fistula—multiple inflow arteries into a nidus pattern with direct artery/arterioles to vein/venulas; type III, arteriolovenular malformation, or nidus-type AVM—multiple arteries/arterioles into an enlarged aneurysmal vein with an enlarged one or more outflow veins; type IV—multiple micro-arteriovenoular connections infiltrating an entire tissue.

There is a higher incidence of AVMs/AVFs in CM-AVM1 (31%) compared with CM-AVM2 (18%) [2]. Intracranial high-flow lesions are more frequent in CM-AVM1 (10%), while the risk is much lower in CM-AVM2 (3%).

CNS or spinal AVMs/AVFs frequently present early in life and may cause seizures, hydrocephalus, headaches, hemorrhage, acute sensorimotor deficits, lower extremity weakness, neurogenic bladder, developmental delay, or high-output cardiac failure [3,4,25,30,31]. Historically, it was believed that if a patient with CM-AVM had normal imaging screens, they would not develop an AVM later in life. However, in 2021 researchers documented the first known case of a de novo intracranial AVM in a six-year-old patient with a RASA1 mutation [32]. Up to half of the reported AVMs/AVFs involve the head and neck [10], but this distribution may be biased by preferential imaging of these regions. Most affected individuals have a single high-flow lesion, though multiple lesions have been reported [18]. AVMs are dynamic entities with potential for progressive dilation, recurrence after incomplete treatment, and regrowth even following apparently complete resection.

Parkes Weber syndrome (PKWS) represents a distinct but overlapping phenotype, defined by a capillary stain, limb overgrowth due to soft tissue and skeletal hyperplasia, and multiple micro-arteriolovenous fistulas [4]. PKWS occurs in both CM-AVM1 and CM-AVM2 [26].

Telangiectasias and Bier spots are characteristic of CM-AVM2, but rare in CM-AVM1. In CM-AVM2, telangiectasias often appear in early childhood, may be punctate or spidery, diffusely distributed, frequently involving the lips, perioral region, trunk, and extremities [4,26]. Bier spots are pinpoint telangiectasias surrounded by a pale halo. Epistaxis is frequent in CM-AVM2, but absent in CM-AVM1 [26].

One of the most severe systemic manifestations is cardiac overload or failure secondary to large shunts, with rare reports of nonimmune hydrops fetalis [33]. Congenital heart defects have been described in a small number of individuals with CM-AVM1 and CM-AVM2, although a causal association remains uncertain [3,11,34,35,36]. Lymphatic anomalies, including lymphedema, chylothorax, and chylous ascites, have been reported in both subtypes, with imaging demonstrating dilated, tortuous lymphatic channels and sluggish flow [3,11,37,38].

An increased risk of tumor development has been suggested but not definitively established. While early reports described a variety of benign and malignant tumors [3], larger series identified only isolated cases of common basal cell carcinoma [10]. It remains uncertain whether tumor incidence exceeds that of the general population.

Finally, a range of additional findings, such as headaches, seizures, hydrocephalus, neurogenic bladder, varicosities, and hemangiomas, have been reported sporadically. Whether these represent primary manifestations of CM-AVM syndrome, secondary consequences of vascular malformations, or coincidental findings remains unknown.

**Table 1:** Characteristics of the two types of CM-AVM syndrome (CM-AVM—capillary malformation-arteriovenous malformation; CNS—central nervous system; HHT—hereditary hemorrhagic telangiectasia) [26,27].

	CM-AVM1	CM-AVM2
Disease causing genes	RASA1	EPHB4
Onset	Early childhood, congenital	Early childhood, congenital
Capillary malformations	+	+
High-flow vascular malformations	30% of patients (10% CNS; rare visceral vascular malformations) [27]	18% of patients (3% CNS vascular malformations) [27]
Telangiectasia	−	+ Infancy/childhood Spidery >> punctate Diffuse
Epistaxis	−	+ Intermittent mild episodes with spontaneous resolution
Bier spots	−	+
Parkes Weber syndrome	7% of patients	7% of patients
Family history	+/−	+/−
Clinical features	Often discrete symptoms	Often discrete symptoms; can mimic HHT

### 3.3. Diagnosis

CM-AVM syndrome remains an underdiagnosed entity, and standardized diagnostic criteria are needed. Suspicion should be high in patients presenting with multifocal capillary malformations (CMs), localized or systemic arteriovenous malformations/fistulas (AVMs/AVFs) involving soft tissue, bone, or the central nervous system, or those exhibiting Parkes Weber syndrome [4].

The diagnosis is currently established in a patient with suggestive clinical findings and a heterozygous pathogenic or likely pathogenic variant in either RASA1 (CM-AVM1) or EPHB4 (CM-AVM2) genes [4]. The terms “pathogenic variants” and “likely pathogenic variants” are synonymous in a clinical setting, meaning that both are considered diagnostic and can be used for clinical decision making [39]. Based on clinical literature, a definitive diagnosis is established by the following criteria [19]:

▪ Primary feature: presence of at least three characteristic CMs (multifocal, small, pink-to-red macules).
▪ Supportive features:
  ○ Radiological or clinical demonstration of an AVM or AVF,
  ○ Positive family history of AVMs or CMs,
  ○ Confirmed pathogenic variant in RASA1 or EPHB4 genes.

The presence of the primary feature in conjunction with any single supportive feature provides a definitive diagnosis. Isolated findings suggest a “probable” diagnosis, requiring molecular confirmation.

Due to the high prevalence of intracranial and spinal lesions, current recommendations include cerebral and medullary MRI/angiography which should be performed in all pediatric patients meeting probable or definite criteria in order to identify asymptomatic high-flow shunts. In the presence of an AVM, rigorous hemodynamic surveillance is mandatory to monitor the local and systemic consequences of high-flow arteriovenous shunting. This longitudinal multimodal assessment should include transcranial Doppler (TCD), CT angiography, echocardiography, and near-infrared spectroscopy (NIRS), to evaluate for potential cerebral hypoperfusion, vascular remodeling, and high-output cardiac failure.

Due to phenotypic overlap, genetic evaluation and counseling should include both the patient and parents for possible EPBH4 and RASA1 mutations. In families with known mutations, fetal ultrasonography is recommended to monitor for in utero AVM development.

Biopsy is not usually recommended as histopathology is non-specific. Microscopic examination typically reveals minimal dermal vascular dilatation delimited by a single layer of endothelial cells, which is also seen in other types of capillary malformations [40]. Dermoscopically, CM-AVM lesions often exhibit a homogeneous brown background reflecting a higher density of mastocytes and potentially leading to an erroneous diagnosis of telangiectatic mastocytosis [41]. Given the non-diagnostic nature of these findings, clinical and molecular diagnosis remain the gold standard.

### 3.4. Differential Diagnosis

In clinical practice, the presentation of multifocal capillary malformations (typically characterized by small, randomly distributed lesions) warrants the inclusion of RASA1 and EPHB4 mutations in the differential diagnosis (Table 2). This diagnostic consideration is important regardless of whether arteriovenous malformations have been identified.

**Table 2:** Differential diagnosis of CM-AVM [27,42].

Differential Diagnosis	Genes Involved	Sustains the Diagnosis	Rules out the Diagnosis
Hereditary hemorrhagic telangiectasia (HHT)	ACVRL1 ENG GDF2 SMAD4	Multiple AVMs that lack intervening capillaries and result in direct connections between arteries and veins	Onset often during adolescence or infancy; telangiectasis are typically punctate/macular and localized rather than diffuse (lips, tongue, nose, face, fingers, ears, conjunctivae); large CMs are not typical; AVMs most often pulmonary, hepatic, cerebral; commonly manifests with spontaneous and recurrent epistaxis episodes, with increasing frequency and severity with age, as well as, cutaneous or mucosal CMs that rupture and bleed after a slight trauma; may associate gastrointestinal bleeding due to telangiectasias in the gastrointestinal mucosa
Sturge–Weber syndrome (SWS)	GNAQ GNA11	Intracranial vascular anomaly localized in the occipital and posterior parietal lobes	Segmental facial cutaneous vascular malformations (port-wine stains) in distribution of the three branches of the trigeminal nerve; leptomeningeal angiomatosis; seizures; high risk of glaucoma
Klippel-Trenaunay syndrome (KTS)	PIK3CA	Capillary malformations and hypertrophy of the related bones and soft tissues; congenital	Vascular malformations are typically low-flow lesions
PTEN hamartoma tumor syndrome	PTEN	Overgrowth and high-flow lesions	Vascular anomalies are usually intramuscular, associated with ectopic fat, severely disrupting tissue architecture; tumor predisposition is more prominent
Multiple cutaneous and mucosal venous malformations (VMCMs)	TEK	Onset frequently in infancy and childhood; cutaneous venous malformations can be mistaken for CMs	Small, soft, compressible, multifocal bluish cutaneous and/or mucosal venous malformations with slow flow on Doppler ultrasound; usually present at birth but new lesions appear with time; small lesions are usually asymptomatic while larger lesions can invade the subcutaneous muscle and cause pain
Hereditary glomuvenous malformations (GVMs)	GLMN	Onset frequently in infancy	Cobble–stone appearance, hard consistency, only partially compressible, painful on palpation; usually not found in mucosa; histopathology—rounded cells (glomus cells) around blood-filled cavities, cells stain positively with smooth muscle α-actin and vimentin

## 4. Discussions

The three cases illustrate a spectrum of vascular disease related to EPHB4 and RASA1 mutations (Table 3). Their evolution—from isolated arteriovenous malformation, through inflammatory endothelial activation with macrophage activation syndrome, to mixed-flow cerebrovascular malformation caused by a dual EPHB4–RASA1 defect—demonstrates that these genes operate within a shared developmental and immunovascular pathway [1,43]. Together, they define a molecular and clinical spectrum ranging from clinically stable arteriovenous malformations to high-flow arteriovenous shunting and immune dysregulation [44,45].

Case 1 presented with a predominantly structural vascular phenotype characterized by cortical hypoperfusion due to chronic steal effect. The patient remained clinically stable under conservative management and imaging surveillance.

Case 2 demonstrated a progressive inflammatory endothelial phenotype, with cerebral hemodynamic deterioration associated with cytokine activation, hyperferritinemia, and hemophagocytic lymphohistiocytosis/macrophage activation syndrome (HLH/MAS). These findings suggest that EPHB4 dysfunction may contribute not only to vascular malformation but also to endothelial-mediated immune dysregulation.

Case 3 represented the most complex phenotype, combining diffuse vascular dysplasia, cerebral vascular anomalies, hydrops fetalis, and mixed-flow malformations in the setting of dual EPHB4 and RASA1 variants. The coexistence of both mutations may explain the overlap between venous dysplasia and arteriovenous shunting.

### 4.1. Imaging Characteristics and Multimodal Evaluatio n

Multimodal neurovascular imaging played a central role in diagnosis and follow-up across all three cases.

In Case 1, Doppler ultrasound served as the earliest diagnostic indicator by detecting vascular anomalies and progressive reduction in cerebral resistive indices. MRI time-of-flight (TOF) imaging further characterized the nidus morphology and venous drainage pattern, allowing differentiation between AVM and vein-of-Galen malformation. Diffusion-weighted imaging (DWI) additionally demonstrated secondary ischemic injury related to vascular steal phenomenon.

In Case 2, transcranial Doppler revealed a marked decline in cerebral resistive indices, indicating severe hemodynamic impairment and vascular autoregulatory dysfunction. The progressive RI decrease correlated with systemic inflammatory activation and vasoplegia.

In Case 3, MRI demonstrated serpiginous subdural and cortical vessels, venous engorgement, calvarial thickening, and diffuse vascular remodeling consistent with long-standing hemodynamic overload. These findings closely resemble imaging patterns previously reported in both EPHB4-related CM-AVM2 and RASA1-related CM-AVM1.

The present cases emphasize the importance of combining vascular Doppler ultrasound, TCD, MRI/MRA, and CT angiography when evaluating suspected mixed-flow cerebrovascular malformations.

### 4.2. Laboratory and Immunological Findings

Case 2 highlighted the potential inflammatory and immunological dimension of EPHB4-associated disease. Progressive cytokine activation, markedly elevated ferritin levels, and increased IL-6 concentrations were associated with severe cerebral hemodynamic deterioration and endothelial dysfunction.

Histopathological confirmation of hemophagocytosis established the diagnosis of secondary HLH/MAS, likely triggered by endothelial injury in the context of EPHB4 mutation. These findings support recent observations suggesting that EPHB4 dysfunction may increase endothelial susceptibility to inflammatory cytokines and impair vascular autoregulation [46].

The drastic reduction in cerebral resistive index (RI < 0.35) paralleled systemic vasoplegia, linking cerebral microvascular dysfunction to cytokine storm physiology. These observations expand the recognized clinical spectrum of EPHB4-related disorders toward inflammatory endothelial disease.

### 4.3. Genetic Findings and Molecular Mechanisms

Both EPHB4 and RASA1 regulate critical pathways involved in vascular morphogenesis and endothelial homeostasis.

EPHB4, through ephrinB2–EPHB4 bidirectional signaling, maintains venous endothelial identity and vascular stability [43]. RASA1 encodes p120-RasGAP, a negative regulator of the RAS/MAPK pathway that limits excessive endothelial proliferation and arteriovenous fusion [1].

Disruption of either pathway destabilizes vascular identity and impairs normal arteriovenous segregation. In Case 3, the coexistence of EPHB4 and RASA1 variants produced a combined phenotype with features of both venous dysplasia and high-flow arteriovenous shunting, supporting the concept of a shared developmental signaling network rather than entirely distinct vascular disorders [45].

Across the three cases, a common pathophysiological pattern emerged:

• Loss of vascular identity through EPHB4–ephrinB2 imbalance
• Abnormal endothelial activation via Ras/MAPK dysregulation [44,45]
• Inflammatory amplification associated with IL-6 and macrophage recruitment
• Progressive vascular remodeling with venous dilatation and autoregulatory failure

### 4.4. Differential Diagnosis and Diagnostic Considerations

The observed phenotypes overlap with several congenital and acquired cerebrovascular disorders and therefore require careful differential diagnosis.

In neonates with high-flow vascular lesions, differentiation from vein-of-Galen aneurysmal malformation is essential and may be achieved through detailed MRI/MRA characterization of venous drainage and nidus architecture. The combined vascular phenotype observed in Case 3 may also mimic Sturge–Weber syndrome because of associated facial capillary malformations, cortical venous dysplasia, and neurological manifestations. However, the absence of cortical calcifications and the presence of pathogenic EPHB4/RASA1 variants favor a genetic vasculopathy rather than sporadic angiogenic dysregulation.

Additional differential diagnoses may include hereditary hemorrhagic telangiectasia, isolated sporadic AVMs, congenital venous malformations, and inflammatory vasculopathies associated with systemic immune activation.

These findings highlight the importance of integrating clinical presentation, multimodal imaging, inflammatory markers, and genetic testing in the diagnostic evaluation of complex pediatric vascular malformations.

### 4.5. Clinical Management and Follow-Up

Management strategies depend on hemodynamic severity, neurological involvement, and inflammatory activity.

In Case 1, conservative management with serial Doppler ultrasound and MRI surveillance was preferred because the patient remained hemodynamically stable. Endovascular embolization or surgical intervention should be reserved for patients with progressive neurological deficits, worsening cerebral hypoperfusion, or cardiac overload.

Case 2 emphasizes the importance of combining neurovascular monitoring with inflammatory markers surveillance, including ferritin, IL-6, complete blood count, and coagulation parameters. Anti-inflammatory or immunomodulatory approaches may warrant consideration in selected cases of EPHB4-associated inflammatory endothelial disease [46].

In Case 3, multimodal imaging follow-up was essential to characterize the evolving mixed-flow hemodynamics and progressive vascular remodeling.

Across all three cases, serial Doppler resistive index measurements appeared to provide an early indicator of cerebral vascular decompensation and may represent a useful noninvasive monitoring tool.

### 4.6. Genetic Counseling and Family Implications

Both EPHB4- and RASA1-related disorders are typically inherited in an autosomal dominant manner with variable expressivity and incomplete penetrance. Identification of pathogenic variants has important implications for family counseling, recurrence risk assessment, and screening of potentially affected relatives.

Early genetic diagnosis may facilitate anticipatory neurovascular surveillance, prenatal counseling, and individualized long-term follow-up strategies, particularly in families with severe or mixed-flow vascular phenotypes.

### 4.7. Proposed Diagnostic and Clinical Approach

Based on the present observations, neonates or infants presenting with cerebrovascular malformations may benefit from the following diagnostic approach:

1. Initial Doppler ultrasound and transcranial Doppler evaluation
2. MRI/MRA characterization of vascular anatomy and hemodynamics
3. Laboratory assessment including ferritin, IL-6, complete blood count, and coagulation profile when inflammatory activation is suspected
4. Genetic testing including EPHB4 and RASA1 analysis
5. Longitudinal neurovascular imaging surveillance and multidisciplinary follow-up [47]
6. Family counseling and consideration of familial genetic screening.

## 5. Conclusions

Maintaining a high index of clinical suspicion when encountering atypical CMs is imperative to establish the diagnosis of capillary malformation–arteriovenous malformation syndrome. This vigilance is necessitated by the risk of life-threatening complications, including high-output cardiac failure or intracranial hemorrhage secondary to high-flow vascular lesions, alongside potential systemic immunological dysfunction.

As clinical data regarding this syndrome expands, a more comprehensive understanding of its phenotypic spectrum will facilitate the refinement of evidence-based screening protocols and management guidelines. The three cases demonstrate that EPHB4 and RASA1 mutations represent points on a vasculo-immunologic spectrum, with a foundation of endothelial instability and aberrant signaling. Understanding this shared molecular mechanism provides new perspectives for precision diagnostics, longitudinal monitoring, and potential targeted therapies in pediatric and congenital vascular malformations.

## Figures and Tables

**Table 3:** Summary of the main characteristics of the three cases—genetic, pathophysiological, clinical, laboratory and imaging aspects.

Case	Genetic Background	Dominant Mechanism	Imaging Phenotype	Clinical and Laboratory Features
1	RASA1 monoallelic variant	High-flow AVM with compensated but chronic cortical hypoperfusion (steal effect)	Slowly progressive decline in cerebral resistive index	Clinically stable
2	EPHB4 + macrophage activation syndrome	Endothelial inflammation, cytokine storm, hemophagocytic activation	Sharp decline in cerebral resistive index (RI < 0.35); hyperemia and vasoplegia on Doppler	Highly elevated ferritin and IL-6 levels, HLH histopathology
3	Combined EPHB4 + RASA1 mutations	Dual pathway dysgenesis (venous + arteriovenous)	Mixed-flow malformation with serpiginous subdural shunts and calvarial thickening	Chronic hyperemia; vascular remodeling

## Data Availability

Not applicable.

## References

[1] Eerola I., Boon L.M., Mulliken J.B., Burrows P.E., Dompmartin A., Watanabe S., Vanwijck R., Vikkula M. (2003). Capillary Malformation-Arteriovenous Malformation, a New Clinical and Genetic Disorder Caused by RASA1 Mutations. Am. J. Hum. Genet..

[2] Amyere M., Revencu N., Helaers R., Pairet E., Baselga E., Cordisco M., Chung W., Dubois J., Lacour J.P., Martorell L. (2017). Germline loss-of-function mutations in EPHB4 cause a second form of capillary malformation-arteriovenous malformation (CM-AVM2) deregulating RAS-MAPK signaling. Circulation.

[3] Revencu N., Boon L.M., Mulliken J.B., Enjolras O., Cordisco M.R., Burrows P.E., Clapuyt P., Hammer F., Dubois J., Baselga E. (2008). Parkes Weber syndrome, vein of galen aneurysmal malformation, and other fast-flow vascular anomalies are caused by RASA1 mutations. Hum. Mutat..

[4] Bayrak-Toydemir P., Stevenson D.A., Adam M.P., Feldman J., Mirzaa G.M. (2011). Capillary Malformation-Arteriovenous Malformation Syndrome.

[5] Bizubac A.M., Fleaca M.A., Herișeanu M.C., Nedelcu C., Bratu A., Marcu V., Filip C., Cîrstoveanu C. (2025). Vein of Galen Malformation—Experience of the Last 13 Years in a Reference Center from South-Eastern Europe. Life.

[6] Nedelcu C., Ionescu N.S., Bizubac A.M., Filip C., Cirstoveanu C. (2025). Trends and Challenges in Noninvasive Hemodynamic Monitoring of Neonates Following Cardiac Surgery: A Narrative Review. Life.

[7] Toma A.I. (2023). Paediatric neurology: Standardization of neonatal assessment in Romania. Enfance.

[8] Revencu N., Fastre E., Ravoet M., Helaers R., Brouillard P., Bisdorff-Bresson A., Chung C.W.T., Gerard M., Dvorakova V., Irvine A.D. (2020). RASA1 mosaic mutations in patients with capillary malformation-arteriovenous malformation. J. Med. Genet..

[9] Wooderchak-Donahue W.L., Johnson P., McDonald J., Blei F., Berenstein A., Sorscher M., Mayer J., Scheuerle A.E., Lewis T., Grimmer J.F. (2018). Expanding the clinical and molecular findings in RASA1 capillary malformation-arteriovenous malformation. Eur. J. Hum. Genet..

[10] Revencu N., Boon L.M., Mendola A., Cordisco M.R., Dubois J., Clapuyt P., Hammer F., Amor D.J., Irvine A.D., Baselga E. (2013). RASA1 Mutations and Associated Phenotypes in 68 Families with Capillary Malformation-Arteriovenous Malformation. Hum. Mutat..

[11] Macmurdo C.F., Wooderchak-Donahue W., Bayrak-Toydemir P., Le J., Wallenstein M.B., Milla C., Teng J.M.C., Bernstein J.A., Stevenson D.A. (2016). RASA1 somatic mutation and variable expressivity in capillary malformation/arteriovenous malformation (CM/AVM) syndrome. Am. J. Med. Genet. A.

[12] Lapinski P.E., Doosti A., Salato V., North P., Burrows P.E., King P.D. (2018). Somatic second hit mutation of RASA1 in vascular endothelial cells in capillary malformation-arteriovenous malformation. Eur. J. Med. Genet..

[13] Cai R., Liu F., Liu Y., Chen H., Lin X. (2018). RASA-1 somatic “second hit” mutation in capillary malformation–arteriovenous malformation. J. Dermatol..

[14] Amyere M., Aerts V., Brouillard P., McIntyre B.A.S., Duhoux F.P., Wassef M., Enjolras O., Mulliken J.B., Devuyst O., Antoine-Poirel H. (2013). Somatic uniparental isodisomy explains multifocality of glomuvenous malformations. Am. J. Hum. Genet..

[15] Akers A.L., Johnson E., Steinberg G.K., Zabramski J.M., Marchuk D.A. (2009). Biallelic somatic and germline mutations in cerebral cavernous malformations (CCMs): Evidence for a two-hit mechanism of CCM pathogenesis. Hum. Mol. Genet..

[16] Pagenstecher A., Stahl S., Sure U., Felbor U. (2009). A two-hit mechanism causes cerebral cavernous malformations: Complete inactivation of CCM1, CCM2 or CCM3 in affected endothelial cells. Hum. Mol. Genet..

[17] Limaye N., Wouters V., Uebelhoer M., Tuominen M., Wirkkala R., Mulliken J.B., Eklund L., Boon L.M., Vikkula M. (2009). Somatic mutations in angiopoietin receptor gene TEK cause solitary and multiple sporadic venous malformations. Nat. Genet..

[18] Grillner P., Söderman M., Holmin S., Rodesch G. (2016). A spectrum of intracranial vascular high-flow arteriovenous shunts in RASA1 mutations. Child’s Nerv. Syst..

[19] Orme C.M., Boyden L.M., Choate K.A., Antaya R.J., King B.A. (2013). Capillary malformation—Arteriovenous malformation syndrome: Review of the literature, proposed diagnostic criteria, and recommendations for management. Pediatr. Dermatol..

[20] Kawasaki J., Aegerter S., Fevurly R.D., Mammoto A., Mammoto T., Sahin M., Mably J.D., Fishman S.J., Chan J. (2014). RASA1 functions in EPHB4 signaling pathway to suppress endothelial mTORC1 activity. J. Clin. Investig..

[21] Aoyagi Y., Schwartz A.W., Li Z., Bai H., Gonzalez L., Lazcano Etchebarne C., Ohashi Y., Kano M., Ho B., Martin K. (2025). Changes in vascular identity during vascular remodeling. JVS. Vasc. Sci..

[22] Tas B., Starnoni D., Smajda S., Vivanti A.J., Adamsbaum C., Eyries M., Melki J., Tawk M., Ozanne A., Revencu N. (2022). Arteriovenous Cerebral High Flow Shunts in Children: From Genotype to Phenotype. Front. Pediatr..

[23] Palermo M., Olivi A., Sturiale C.L. (2025). Capillary malformation–arteriovenous malformation syndrome (CM-AVM): A systematic review of cerebrovascular manifestations. Child’s Nerv. Syst..

[24] Wooderchak-Donahue W.L., Akay G., Whitehead K., Briggs E., Stevenson D.A., O’fallon B., Velinder M., Farrell A., Shen W., Bedoukian E. (2019). Phenotype of CM-AVM2 caused by variants in EPHB4: How much overlap with hereditary hemorrhagic telangiectasia (HHT)?. Genet. Med..

[25] Burrows P.E. (2017). Angioarchitecture of Hereditary Arteriovenous Malformations. Semin. Interv. Radiol..

[26] Cordisco M.R., El-Feghaly J., Prezzano J.C., Lanöel A., Torres N., Persico S., Requejo F., Sierre S., Fiandrino M.J., Luna L. (2022). Capillary Malformation-Arteriovenous Malformation Type 2, A Report of 6 Cases and Main Differential Diagnosis. J. Vasc. Anom..

[27] Brix A.T.H., Tørring P.M., Bygum A. (2022). Capillary Malformation-arteriovenous Malformation Type 2: A Case Report and Review. Acta Derm.-Venereol..

[28] Rodríguez Bandera A.I., Feito Rodríguez M., Chiloeches Fernández C., Stewart N., Valdivielso-Ramos M. (2020). Role of colour-Doppler high-frequency ultrasonography in capillary malformation-arteriovenous malformation syndrome: A case series. Australas. J. Dermatol..

[29] Yakes W.F., Vogelzang R.L., Ivancev K., Yakes A.M. (2017). New arteriographic classification of AVM based on the yakes classification system. Congenital Vascular Malformations: A Comprehensive Review of Current Management.

[30] Wooderchak-Donahue W., Stevenson D.A., McDonald J., Grimmer J.F., Gedge F., Bayrak-Toydemir Pinar P. (2012). RASA1 analysis: Clinical and molecular findings in a series of consecutive cases. Eur. J. Med. Genet..

[31] Thiex R., Mulliken J.B., Revencu N., Boon L.M., Burrows P.E., Cordisco M., Dwight Y., Smith E.R., Vikkula M., Orbach D.B. (2010). A Novel Association between RASA1 Mutations and Spinal Arteriovenous Anomalies. Am. J. Neuroradiol..

[32] Ryu B., Sato S., Mochizuki T., Inoue T., Okada Y., Niimi Y. (2021). De novo intracranial arteriovenous malformation development after endovascular treatment for a pial arteriovenous fistula in capillary malformation-arteriovenous malformation syndrome. Interv. Neuroradiol..

[33] Overcash R.T., Gibu C.K., Jones M.C., Ramos G.A., Andreasen T.S. (2015). Maternal and fetal capillary malformation-arteriovenous malformation (CM-AVM) due to a novel RASA1 mutation presenting with prenatal non-immune hydrops fetalis. Am. J. Med. Genet. A.

[34] Zheng Y., Peng Y., Zhang S., Li L., Peng Y., Yin Q. (2019). Capillary Malformation–Arteriovenous Malformation Combined Alagille Syndrome in a Patient With Double Gene Variations of RASA1 and NOTCH2. Front. Genet..

[35] Martin-Almedina S., Ogmen K., Sackey E., Grigoriadis D., Karapouliou C., Nadarajah N., Ebbing C., Lord J., Mellis R., Kortuem F. (2021). Janus-faced EPHB4-associated disorders: Novel pathogenic variants and unreported intrafamilial overlapping phenotypes. Genet. Med..

[36] Chennappan S., Kontaridis M.I. (2026). RASopathies in Cardiac Disease. Annu. Rev. Med..

[37] de Wijn R.S., Oduber C.E.U., Breugem C.C., Alders M., Hennekam R.C.M., van der Horst C.M.A.M. (2012). Phenotypic variability in a family with capillary malformations caused by a mutation in the RASA1 gene. Eur. J. Med. Genet..

[38] Burrows P.E., Gonzalez-Garay M.L., Rasmussen J.C., Aldrich M.B., Guilliod R., Maus E.A., Fife C.E., Kwon S., Lapinski P.E., King P.D. (2013). Lymphatic abnormalities are associated with RASA1 gene mutations in mouse and man. Proc. Natl. Acad. Sci. USA.

[39] Richards S., Aziz N., Bale S., Bick D., Das S., Gastier-Foster J., Grody W.W., Hegde M., Lyon E., Spector E. (2015). Standards and guidelines for the interpretation of sequence variants: A joint consensus recommendation of the American College of Medical Genetics and Genomics and the Association for Molecular Pathology. Genet. Med..

[40] Gourier G., Audebert-Bellanger S., Vourc’h P., Fraitag S., L’Hérondelle K., Labouche A., Misery L., Abasq-Thomas C. (2018). Multiple capillary malformations of progressive onset: Capillary malformation–arteriovenous malformation syndrome (CM-AVM). Ann. Dermatol. Venereol..

[41] Gandon C., Bonniaud B., Collet E., Dalac S., Jeudy G., Vabres P. (2016). A Typical Vascular and Pigmentary Dermoscopic Pattern of Capillary Malformations in Capillary Malformation–Arteriovenous Malformation Syndrome: Report of Four Cases. Pediatr. Dermatol..

[42] Ustaszewski A., Janowska-Głowacka J., Wołyńska K., Pietrzak A., Badura-Stronka M. (2021). Genetic syndromes with vascular malformations—Update on molecular background and diagnostics. Arch. Med. Sci..

[43] Li D., Wenger T.L., Seiler C., March M.E., Gutierrez-Uzquiza A., Kao C., Bhoj E., Tian L., Rosenbach M., Liu Y. (2018). Pathogenic variant in EPHB4 results in central conducting lymphatic anomaly. Hum. Mol. Genet..

[44] Xue J., Zhang Z., Sun Y., Jin D., Guo L., Li X., Zhao D., Feng X., Qi W., Zhu H. (2023). Research Progress and Molecular Mechanisms of Endothelial Cells Inflammation in Vascular-Related Diseases. J. Inflamm. Res..

[45] Wang S., Deng X., Wu Y., Wu Y., Zhou S., Yang J., Huang Y. (2023). Understanding the pathogenesis of brain arteriovenous malformation: Genetic variations, epigenetics, signaling pathways, and immune inflammation. Hum. Genet..

[46] Jordan M.B., Allen C.E., Greenberg J., Henry M., Hermiston M.L., Kumar A., Hines M., Eckstein O., Ladisch S., Nichols K.E. (2019). Challenges in the diagnosis of hemophagocytic lymphohistiocytosis: Recommendations from the North American Consortium for Histiocytosis (NACHO). Pediatr. Blood Cancer.

[47] Toma A.I., Dima V., Rusu L., Nemeș A.F., Gonț B.F., Arghirescu A., Necula A., Fieraru A., Stoiciu R., Andrășoaie L. (2025). Cerebral Ultrasound at Term-Equivalent Age: Correlations with Neuro-Motor Outcomes at 12–24 Months Corrected Age. Children.

